# Performance Analysis of GPS/BDS Dual/Triple-Frequency Network RTK in Urban Areas: A Case Study in Hong Kong

**DOI:** 10.3390/s18082437

**Published:** 2018-07-26

**Authors:** Ying Xu, Wu Chen

**Affiliations:** 1 College of Geomatics, Shandong University of Science and Technology, Qingdao 266590, China; 2 Department of Land Surveying and Geo-Informatics, The Hong Kong Polytechnic University, Hong Kong, China; wu.chen@polyu.edu.hk

**Keywords:** GPS/BDS, Network RTK, dual/triple-frequency, ambiguity successfully fixed rate, urban areas

## Abstract

Network Real Time Kinematic (NRTK) positioning with instantaneous ambiguity resolution (AR) is currently one of the most popular techniques for real-time precise positioning using Global Navigation Satellite Systems (GNSS) carrier phase observations. Although NRTK has been successfully applied in many fields in surveying and navigation, the initialization speed, accuracy, and ambiguity successfully fixed rate of NRTK in urban areas (Hong Kong, for instance) would be significantly affected by blocked satellite signals. To address these problems and analyze the performance of GPS/BDS dual/triple-frequency NRTK in urban areas, we developed a new Hong Kong GNSS Network RTK Service Platform. Based on this platform, the performance of NRTK in urban areas was examined through a series of experiments. The results showed that: (1) The initialization time of the NRTK varied with the number of the visible satellite and the quality of the observation. (2) Centimeter-level NRTK service could be provided for users over Hong Kong using the Hong Kong GNSS Network RTK Service Platform. (3) In urban areas, GPS/BDS NRTK services for static, walking, and driving users significantly improved the ambiguity successfully fixed rate of the NRTK service when compared with that using the GPS signal alone. The NRTK ambiguity successfully fixed rate in Hong Kong was better than 99% in good environment. In typical urban environment, the RTK ambiguity successfully fixed rate with GPS/BDS was 33.4–72.4%, which was about 12.7–32.4% with GPS only. (4) BDS triple-frequency observation improved the initialization speed and positioning accuracy of RTK in Hong Kong.

## 1. Introduction

Baseline processing based on Global Navigation Satellite Systems (GNSS) differenced carrier phases observation is a standard high-accuracy post-positioning technique. However, various high accuracy positioning tasks need real-time operations. Traditional Real Time Kinematic (RTK) was developed in the mid-1990s, which provided cm-level accuracy positioning in real-time based on GNSS measurements [1]. It involved one reference receiver that transmits raw measurements or observation corrections to a rover receiver via some sort of data communication link (e.g., VHF or UHF radio, and cellular telephone). To provide positioning services to larger region, multiple reference stations have to be set up and maintained, and the development of Network RTK (NRTK) resulted in tremendous reduction of the investment costs, which serves as a prerequisite for starting an RTK positioning service [1]. Compared with traditional RTK, NRTK has many other advantages: capable of modeling GPS errors over the entire network area, increasing mobility and efficiency, reducing initialization times for rovers, extending surveying range, capability of supporting multiple users and applications, and continuous operation. Continuously Operating Reference Stations System (CORS) is the fundamental infrastructure for NRTK. In the United States, the National Geodetic Survey (NGS) manages a network of CORS of more than 2000 stations [2]. The Canadian Active Control System (CACS) provides improved GPS positioning capability for the Canadian surveying and geophysical community as well as for other spatial referencing needs. By mid-2015, there were 102 sites in CACS [3]. In Australia, GNSS data of approximately 100 stations from the Australian Regional GPS Network (ARGN), South Pacific Regional GNSS Network (SPRGN), and the AuScope Network are collected by Geoscience Australia [4,5]. A Satellite Positioning Service of the Satellite Positioning Service of the German National Survey (SAPOS) is operated in Germany. It consists of over 270 reference stations with mean station distances of 25–60 km [6]. The Geospatial Information Authority of Japan (GSI) operates a nationwide GPS observation array, GEONET. Over 1200 GEONET stations are operated with real-time data transmission and a high sampling rate mode. The construction of CORS in China began at the end of 20th century and the development gradually expanded from a city-based system to a nationwide system. In China, different administrations and provinces have built over 4000 reference stations. One of the major national networks in China is the Crustal Movement Observation Network of China, which includes 260 reference stations [7]. It is a network project for comprehensive observations of Earth Sciences, which employs the GNSS system and other complementary space and precise observation technologies to monitor the real-time dynamic changes of the continental tectonic environment and explore influences of these changes on resources, environment, and disasters.

Besides CORS, the NRTK software/platforms also reflect the development of the NRTK. The most commonly used commercial NRTK software/platforms are Trimble Pivot Platform of Trimble, Spider of Leica, and GNNET-RTK of GEO++. Trimble pivot is the fourth generation of CORS system software from Trimble, which is designed to connect a large number of reference stations to become a network correction number, ideal for high-precision positioning. GNSS Spider is a highly integrated software suite for the central control and operation of a single reference station or reference station network. The Spider v5.2 series software fully supports China’s independent research and development of the Beidou satellite navigation system (BDS). GNNET-RTK of GEO++ is able to achieve accuracies of a few millimeters by using antenna and multipath calibrations. However, GNNET-RTK is only currently suitable for GPS + GLONASS. In addition to the above-mentioned commercial NRTK software/platforms, universities and research institutions have also developed their own NRTK software such as the Multi-Purpose GPS Processing Software (MPGPSTM) of The Ohio State University, the software for Multiple Reference stations real-time kinematic GPS application (MultiRef™) by the University of Calgary, and the PowerNetwork of Wuhan University [8]. The network correction modes that these software packages support are different from each other. Based on SSR (State Space Representation) technology, developed and promoted by Geo++, all of today’s GNSS network correction modes (FKP + VRS/PRS + MAC + SSR) are effectively supported by Geo++. The NRTK software of Trimble mainly supports VRS (Virtual Reference Station), and Leica uses the MAC (Master Auxiliary Corrections) technology. The PowerNetwork of Wuhan University applies the modified combined bias interpolation method.

**Figure 1:**
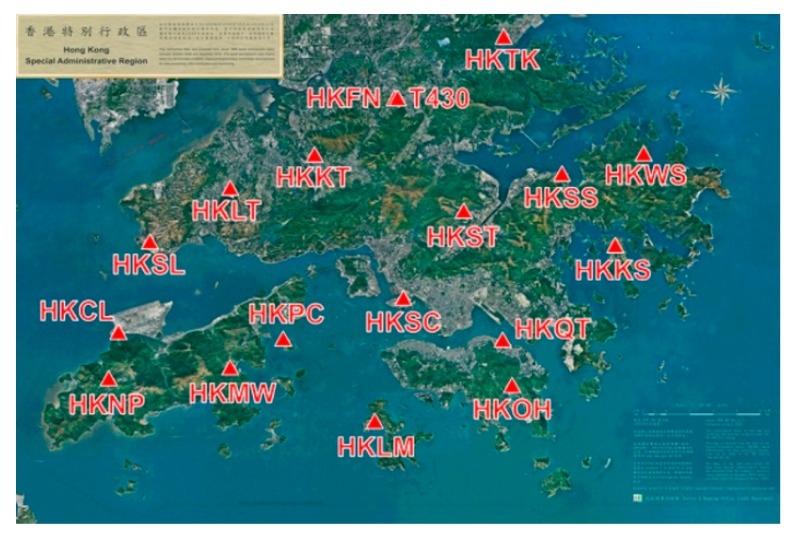
Distribution of Hong Kong SatRef.

Although NRTK has been successfully applied in civilian and military fields, the performance of NRTK in urban areas is affected by many factors. In Hong Kong, for instance, there are too many tall buildings and narrow streets, which will block satellite signals and, therefore, the availability of GPS positioning in urban Hong Kong is extremely low. For example, in the streets of Wanchai, the availability of GPS positioning is only 7% and the extremely large multipath of GPS signals greatly reduces the accuracy of GPS positioning in Hong Kong [9,10]. Hong Kong Satellite Positioning Reference Station Network (SatRef) is a local satellite positioning system established by the Survey and Mapping Office of Lands Department of Hong Kong. The network consists of 18 continuously operating reference stations evenly distributed in Hong Kong (Figure 1). Although SatRef has been successfully applied in surveying applications, there are still some problems that need to be solved. For example, the initialization speed, ambiguity successfully fixed rate, and accuracy of NRTK may sometimes be affected by blocked satellite signals and high ionospheric effects.

Fortunately, GNSS are evolving to a new era. The US GPS system is currently improving through the GPS modernization program. BDS with a global coverage will be completed by 2020 [11]. Among the new GNSS, GPS introduces the L5 signal at 1176.45 MHz in addition to the current L1 at 1575.42 MHz and L2 at 1227.6 MHz, while the Chinese BDS navigation satellite system operates in three frequency bands: 1561.098 MHz; 1207.14 MHz; and 1268.52 MHz. The multiple-constellation and multiple-frequency GNSS data will bring great benefit to the NRTK in urban areas. Most research [12,13,14,15,16,17] has shown that the centimeter level of positioning accuracy can be achieved in a very short initialization time using triple-frequency observations. The first BDS results outside the Chinese mainland were reported by Montenbruck et al., Steigenberger et al., and Nadarajah et al. [18,19,20,21]. Teunissen et al. analyzed the instantaneous RTK positioning capabilities of a combined GPS + BDS system for cut-off elevation angles ranging between 10° and 40° in terms of the ADOP, the bootstrapped success rate, and the positioning precision. Test results showed that the GPS/BDS combination had good high-precision positioning performance for up to a 40° cut-off elevation [20]. The result is important as such a measurement set-up will significantly increase the GNSS applicability in constrained environments such as in urban canyons or when a low-elevation multipath is present. However, only a few RTK tests have been conducted in the urban environment. The GPS/BDS dual/triple frequency NRTK performance assessment in urban areas especially in Hong Kong is limited.

In this study, we developed a new NRTK server platform. This platform integrates multiple-constellation and multiple-frequency GNSS data to support reliable NRTK operation in Hong Kong. It is worth mentioning that the BDS observation collected in our study was from the BDS-2 system [19]. Based on this platform, the performance of NRTK in urban areas was examined using a series of experiments. In this paper, we first introduce the development of CORS, NRTK software, and the multiple-constellation and multiple-frequency GNSS, and discuss the issues that affect the performance of NRTK in urban areas. Second, the functions and key technology of the Hong Kong GNSS Network RTK Service Platform are discussed. Third, the ambiguity resolution (AR) technique for triple-frequency signals used in this study is presented. Initialization time test, positioning accuracy test, ambiguity successfully fixed rate test and the triple-frequency NRTK test were carried out to assess the NRTK performance in Hong Kong. Finally, we present our conclusions.

## 2. Hong Kong GNSS Network RTK Service Platform

The Hong Kong GNSS Network RTK Service Platform was designed as a spatial positioning service platform using VRS technology. It was able to provide a real-time centimeter-level positioning service using GNSS dual-frequency and multiple-frequency signals for NRTK users in Hong Kong. The Hong Kong GNSS Network RTK Service Platform’s workflow was similar to the workflow of the standard NRTK [22,23,24,25]. It decodes the original data from the server into code and carrier phase observation and preprocesses them after the satellite positions are calculated with the broadcast ephemeris. Then, it builds the single- and double-difference observation equations and initials the double difference ambiguity or passes the double difference ambiguity of the last epoch for the baseline between the reference stations. It estimates the double difference tropospheric delay and ionospheric delay. Finally, it receives approximate coordinates of users, generates VRS corrections, and transfers them to users. Users apply the VRS data and simple double-difference to achieve the positioning service.

The Hong Kong GNSS Network RTK Service Platform implements the following key functions:

1. Decode the data from Hong Kong SatRef.
2. Store observations and ephemeris in the format of RINEX.
3. Correct the antenna phase center offset.
4. Detect the outlier of the observation and repair cycle slips.
5. Fix the double difference ambiguity between the reference stations.
6. Estimate the double difference tropospheric delay and ionospheric delay.
  The tropospheric delay can be divided into two parts: the hydrostatic delay and the non- hydrostatic or wet delay. We declined the first form by using the empirical model. For the wet delay, we estimated it together with ambiguities in the data processing. Once the ambiguity was fixed correctly, the ionospheric delay could be estimated using the following observation combination,
  (1) $$\Delta \nabla \delta_{I_{1}}=\left(\Delta \nabla \Phi_{1}-\Delta \nabla \Phi_{2}+\lambda_{1}\Delta \nabla {\hat{N}}_{1}-\lambda_{2}\Delta \nabla {\hat{N}}_{2}\right)/\left(\beta_{2}-\beta_{1}\right)$$
  where $\beta_{i}$ is the ionospheric scale factor defined with respect to the first-order ionospheric delay on the L1 carrier ($\Delta \nabla \delta_{I_{1}}$); ∆∇Φ is the double difference carrier phase observation; $\Delta \nabla \hat{N}$ is the fixed double difference ambiguity; and λ is the wavelength.
7. Generate VRS corrections.
  VRS corrections include the tropospheric delay, ionospheric delay, orbit error, etc. of the VRS observation.
8. Transfer RTCM (Radio Technical Commission for Maritime Services) data to users.
  In this study, the RTCM-3 MSM4 (Multiple Signal Message 4) was applied. This generated RTCM data were sent to the user through a wireless connection, using the Networked Transport of RTCM via the Internet Protocol (NTRIP).
9. Allow the processing of data from many GNSS (GPS, BDS, GLONASS, etc.).
10. Provide centimeter-level accuracy NRTK service.

In addition, the Hong Kong GNSS Network RTK Service Platform supports the triple-frequency GNSS NRTK, and its ambiguity resolution method will be discussed in the next section.

## 3. Ambiguity Resolution for Triple-Frequency Signals

For the AR of the triple-frequency signals, the definitions of the virtual signals and related parameters should first be given. The general form of the GNSS linear carrier phase and pseudo-range observation combination equations of three fundamental signals can be generally formulated as [16]

(2) $$\;P_{\left(i,j,k\right)}=\frac{i\cdot f_{1}\cdot P_{1}+j\cdot f_{2}\cdot P_{2}+k\cdot f_{3}\cdot P_{3}}{i\cdot f_{1}+j\cdot f_{2}+k\cdot f_{3}}$$

(3) $$\phi_{\left(i,j,k\right)}=\frac{i\cdot f_{1}\cdot \phi_{1}+j\cdot f_{2}\cdot \phi_{2}+k\cdot f_{3}\cdot \phi_{3}}{i\cdot f_{1}+j\cdot f_{2}+k\cdot f_{3}}$$

(4) $$\varphi_{\left(i,j,k\right)}=i\cdot \varphi_{1}+j\cdot \varphi_{2}+k\cdot \varphi_{3}$$

where $P_{\left(i,j,k\right)}$ and $\phi_{\left(i,j,k\right)}$ are the pseudo-range and carrier phase combination in meters, respectively, and $\varphi_{\left(i,j,k\right)}$ is the phase combination in cycles. *i, j,* and *k* are the combination coefficients, which are integers. $P_{i}$ and $\phi_{i}$ are the pseudo-range and phase measurements in meters, and $f_{i}$ is the frequency of the carrier phase. $\varphi_{i}$ is the phase measurement in cycles. The corresponding virtual frequency, wavelength and the ambiguity of the observation combination are

(5) $$f_{\left(i,j,k\right)}=i\cdot f_{1}+j\cdot f_{2}+k\cdot f_{3}$$

(6) $$\lambda_{\left(i,j,k\right)}=c/f_{\left(i,j,k\right)}$$

(7) $$N_{\left(i,j,k\right)}=i\cdot N_{1}+j\cdot N_{2}+k\cdot N_{3}$$

where c denotes the light speed. $N_{i}$ is the ambiguity of the triple-frequency carrier phase measurement.

The virtual double difference pseudo-range and phase signals can be described as

(8) $$\Delta \nabla P_{\left(i,j,k\right)}=\Delta \nabla \rho +\Delta \nabla \delta_{trop}+\beta_{\left(i,j,k\right)}\cdot \Delta \nabla \delta_{I_{1}}+\varepsilon_{\Delta \nabla P_{\left(i,j,k\right)}}$$

(9) $$\Delta \nabla \phi_{\left(i,j,k\right)}=\Delta \nabla \rho +\Delta \nabla \delta_{trop}-\beta_{\left(i,j,k\right)}\cdot \Delta \nabla \delta_{I_{1}}-\lambda_{\left(i,j,k\right)}\cdot \Delta \nabla N_{\left(i,j,k\right)}+\varepsilon_{\Delta \nabla \phi_{\left(i,j,k\right)}}$$

where Δ∇ρ is the double difference geometric distance between the satellite and receiver,$\Delta \nabla \delta_{trop}$ is the double difference tropospheric delay, $\beta_{\left(i,j,k\right)}$ is the ionospheric scale factor defined with respect to the first-order ionopheric delay on the L1 carrier ($\Delta \nabla \delta_{I_{1}}$), and $\varepsilon_{\Delta \nabla P_{\left(i,j,k\right)}}$ and $\varepsilon_{\Delta \nabla \phi_{\left(i,j,k\right)}}$ are the pseudo-range and phase observation noise of the triple-frequency combination, respectively.

Theoretically, there are infinite choices of linear integer combinations, and three of them are linearly independent. For ambiguity resolution purposes, two optimal combinations of extra-wide lane (λ ≥ 2.93 m, EWL) and wide lane (0.75 m ≤ λ < 2.93 m, WL) should first be selected. Generally, the optimal combinations proposed by different researchers are often based on the smallest ionospheric scale factor or largest wavelength-to-noise ratio. In this study, the $\phi_{\left(0,1,-1\right)}$, which has been the straightforward choice, was selected as the first EWL signal [16]. The observation combination with the minimal or near minimal first-order ionospheric scale factor ($\beta_{\left(i,j,k\right)}$) was chosen as the second best EWL/WL observation in this study, which was $\phi_{\left(1,-6,5\right)}$ for GPS and $\phi_{\left(1,-5,4\right)}$ for BDS [16].

Three/Multiple Carrier Ambiguity Resolution (TCAR/MCAR) and Cascading Integer Resolution (CIR) are typical three/multiple-carrier ambiguity resolution method proposed by Forssell et al. [26], Vollath et al. [27], De Jonge et al. [28] and Hatch et al. [29]. The integer estimation principles of TCAR and CIR are both examples of integer bootstrapping. The integer ambiguity of the observation can generally be determined by rounding the float ones to its nearest integer values. However, these methods are biased by the residual ionospheric delay [30,31]. Following these studies, a large amount of work has been carried out on the TCAR/CIR or modified TCAR/CIR methods. Feng et al. [16] resolved the ambiguities of the optimized virtual signals in a three-step procedure. Feng and Li [32] used both geometry-based and geometry-free TCAR model to process the ambiguity resolution. A geometry-free and ionosphere-free for distance-independent reliable TCAR method was imposed in 2010 by Li et al. [33], which was free from both ionospheric effects and geometric terms. Ji et al. [14] presented an improved CAR method which included the advantages of both integer least-squares (ILS) and CAR. Tang et al. [34] proposed a modified stepwise AR method based on the TCAR. Teunissen et al. [30,31]compared the performance of LAMBDA and TCAR/CIR when they were applied to the multiple-frequency geometry-free case and the multiple-frequency geometry-based cases. For the Geometry-free case, TCAR and CIR ambiguity resolution perform as well as The LAMBDA method. For Geometry-based case, although the reliability of LAMBDA is generally better than that of TCAR and CID, the LAMBDA is computationally more intensive. As a result, we combined the TCAR and LAMBDA method to fix the ambiguity for triple-frequency signals. Research has shown that the ionospheric variability in low-latitude regions is much greater than that in mid-latitude areas [35,36,37]. During ionosphere storms, the ionospheric delay for a 10 km baseline can reach tens of meters. As a result, the double difference ionospheric delay residual cannot be ignored or well modeled even for the baseline of 15–30 km in Hong Kong. In this study, we tried to fix the ambiguity of the triple-frequency signals for baselines between reference stations in three steps without the effect of the ionospheric delay. The whole process was:

Step 1. We fixed the ambiguity of $\Delta \nabla \phi_{\left(0,1,-1\right)}$ using the geometry-free method as suggested by most authors (Equation (10)), which is free of ionospheric effects and nearly minimally affected by code noise [16].

(10) $$\Delta \nabla N_{\left(0,1,-1\right)}=Round\left(\frac{\Delta \nabla P_{\left(0,1,1\right)}-\Delta \nabla \phi_{\left(0,1,-1\right)}}{\lambda_{\left(0,1,-1\right)}}\right)$$

Step 2. We formed the double difference ionosphere-free (IF) code observation ($\Delta \nabla P_{IF}$) first, and then computed the $\Delta \nabla \delta_{I_{1}}$ using the code observation *(*$\Delta \nabla P_{1},\Delta \nabla P_{2}$) with Equation (11). Since the first-order ionospheric scale factor of the second best EWL/WL was especially small ($\beta_{\left(1,-6.5\right)}=0.0774$, for instance), the estimation error due to the code observation in Equation (11) only showed limited impact on the ambiguity resolution [33]. Equation (12) was applied to compute the float ambiguity of the second best EWL/WL ($\Delta \nabla \phi_{\left(1,-6,5\right)}$, for instance).

(11) $$\Delta \nabla \delta_{I_{1}}=\frac{f_{2}^{2}}{f_{1}^{2}-f_{2}^{2}}\left(\Delta \nabla P_{2}-\Delta \nabla P_{1}\right)$$

(12) $$\left[\begin{array}{c} \Delta \nabla P_{IF}\\ \Delta \nabla \phi_{\left(1,-6,5\right)}+\beta_{\left(1,-6.5\right)}\cdot \Delta \nabla \delta_{I_{1}} \end{array}\right]=\left[\begin{array}{cc} A & 0\\ A & -\Delta \nabla \lambda_{\left(1,-6,5\right)} \end{array}\right]\left[\begin{array}{c} \delta X\\ \Delta \nabla N_{\left(1,-6,5\right)} \end{array}\right]+\left[\begin{array}{c} \varepsilon_{\Delta \nabla P_{IF}}\\ \varepsilon_{\Delta \nabla \phi_{\left(1,-6,5\right)}} \end{array}\right]$$

where δX is the vector of unknown parameters of the baseline components and A is the baseline design matrix.

Step 3. The unknown parameter of ionospheric delay was involved. The float ambiguity of $\Delta \nabla \phi_{1}$ was computed using Equation (13). The LAMBDA method was used to fix the ambiguity in Steps 2 and 3.

(13) $$\left[\begin{array}{c} \begin{array}{c} \Delta \nabla \phi_{\left(0,1,-1\right)}+\lambda_{\left(0,1,-1\right)}\Delta \nabla N_{\left(0,1,-1\right)}\\ \Delta \nabla \phi_{\left(1,-6,5\right)}+\lambda_{\left(1,-6,5\right)}\Delta \nabla N_{\left(1,-6,5\right)} \end{array}\\ \Delta \nabla \phi_{1} \end{array}\right]=\left[\begin{array}{ccc} A & -\beta_{\left(0,1,-1\right)} & 0\\ A & -\beta_{\left(1,-6,5\right)} & 0\\ A & -\beta_{1} & -\lambda_{1} \end{array}\right]\left[\begin{array}{c} \delta X\\ \Delta \nabla \delta_{I_{1}}\\ \Delta \nabla N_{1} \end{array}\right]+\left[\begin{array}{c} \begin{array}{c} \varepsilon_{\Delta \nabla \phi_{\left(0,1,-1\right)}}\\ \varepsilon_{\Delta \nabla \phi_{\left(1,-6,5\right)}} \end{array}\\ \varepsilon_{\Delta \nabla \phi_{1}} \end{array}\right]$$

The R-ratio test has been developed for decades with several improved versions [38,39]. In this research, we used the R-ratio test with the definition shown in Equation (14) for ambiguity validation. Let the float ambiguity vector and its variance matrix be given as $\hat{a}$ and $Q_{\hat{a}\hat{a}}$, respectively. Furthermore, let $\overset{ˇ}{a}$ be the integer least square solution, i.e., the integer minimizer of $q\left(a\right)={\left(\hat{a}-a\right)}^{T}Q_{\hat{a}\hat{a}}{}^{-1}\left(\hat{a}-a\right)$, and let ${\overset{ˇ}{a}}^{\prime }$ be the integer vector that returns the second smallest value of the quadratic form q(a), and the equation of the ratio-test can be written as:

(14) $$\mathrm{Accept}\;\overset{ˇ}{a}\;\mathrm{if}\;\frac{q\left({\overset{ˇ}{a}}^{\prime }\right)}{q\left(\overset{ˇ}{a}\right)}\geq c$$

In this research, the threshold value was set to 2.5 [40].

## 4. Experiment and Analysis

To assess the NRTK performance in urban areas in the aspects of initialization time, positioning accuracy, ambiguity successfully fixed rate, and the benefits of the triple-frequency observation, a series of experiments using the Hong Kong GNSS Network RTK Service Platform were carried out.

### 4.1. Initialization Time Test

**Figure 2:**
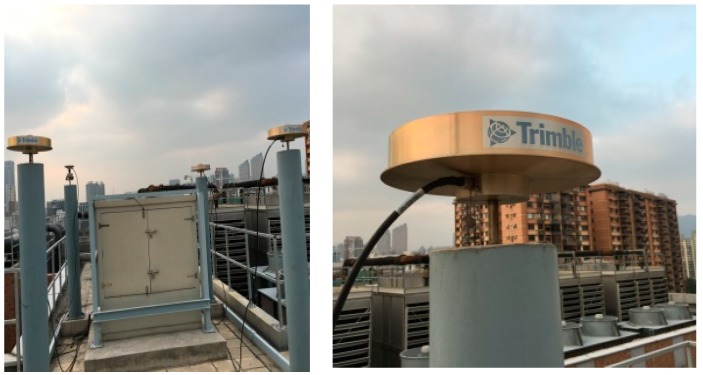
Environment of the station at POLYU.

**Figure 3:**
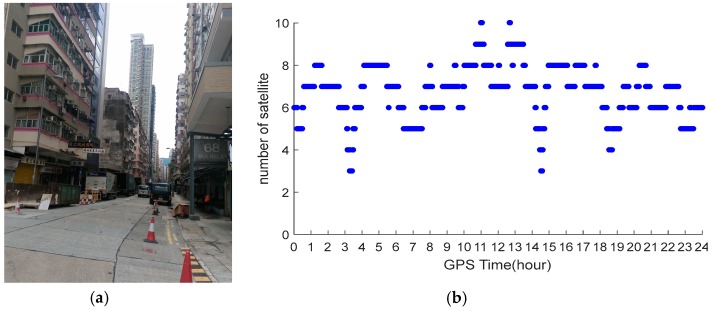
Environment (**a**); and number of satellites (**b**) of the station in urban environment.

To test the initialization time of the NRTK in Hong Kong, experiments were carried out on two stations on 22 June 2016. One of them was located in a good environment and the other one was located in standard urban environment. The reason we designed the experiment in relatively good environment was that we wanted to compare the experiment results with that of the experiment conducted in standard urban environment. The former station was on the roof of Z block at Hong Kong Polytechnic University (POLYU). Figure 2 shows its environment. The latter station was placed on one road where tall buildings along the road significantly occluded the GNSS signal. Figure 3 shows the environment and number of visible GPS/BDS satellites of this station during the whole experimental period. The sample interval was one second. Due to the interruption, the continuity of the GNSS signals is weak. Generally, only 3–10 GPS/BDS satellites are visible, including five BDS GEO (Geostationary Earth Orbit) satellites, and the average number of visible satellites is 4.8. The distance between the stations to the nearest reference stations are 18.2 km and 18.5 km, respectively.

**Figure 4:**
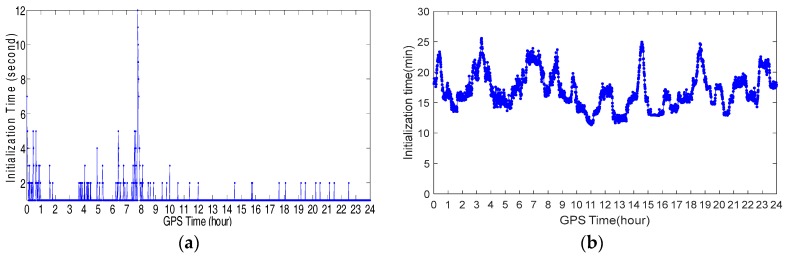
Initialization time of the station at POLYU (**a**) and urban environment (**b**).

GPS data were processed using the correction from the Hong Kong GNSS Network RTK Service Platform. In the data processing, we started from every minute in the dataset until all ambiguities were fixed to their integers. Figure 4a,b shows the initialization time of the stations located at POLYU and in urban environment, respectively. From these figures, we can see that only 1–12 s were necessary for the initialization of the station at POLYU. The initialization time of the station in the complex environment was significantly longer than that of the other stations. The mean time required for this station was 16.8 min. When the number of the visible satellites decreased, the initialization time would increase. Significant multipath affects the initialization of the station located in complex environment. Comparing the test results, we found that the initialization time of the station varied with the number of visible satellites and the quality of the observation.

### 4.2. Positioning Accuracy Test

**Figure 5:**
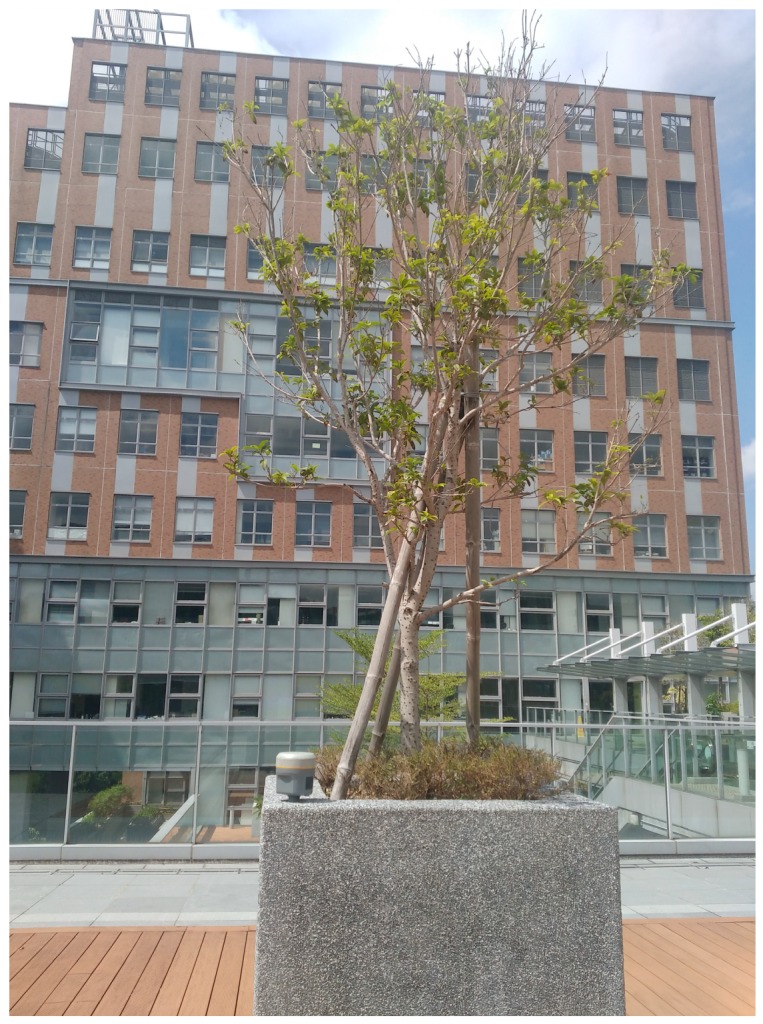
Environment of the station at POLYU.

**Figure 6:**
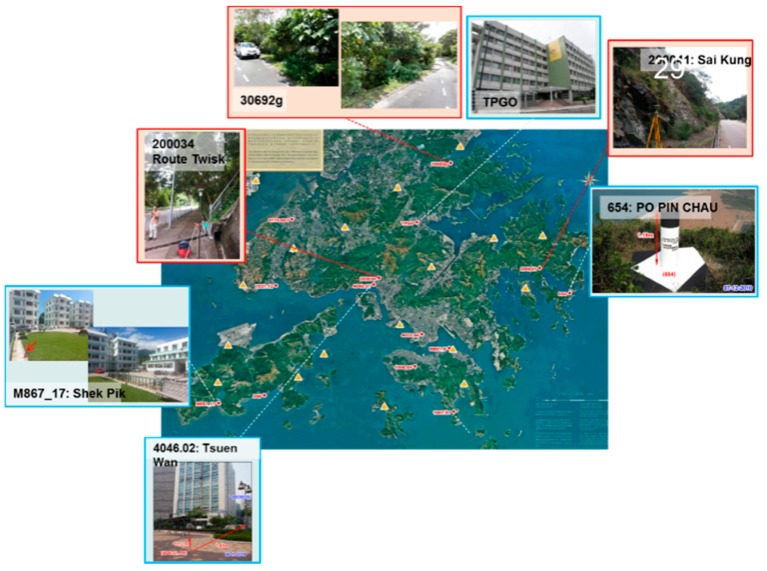
Location and environment of the seven test points.

To analyze the NRTK positioning accuracy performance in Hong Kong, we set one station on the sixth floor platform of Z core in POLYU on 20 July 2016. Figure 5 shows the test environment. The experimental station was located in the terrace garden of the sixth floor where trees and a wall would shelter the GNSS satellites signals. Other positioning accuracy tests were carried out on seven test points evenly distributed in Hong Kong. The location and environment of these test points are shown in Figure 6. The observation conditions of the four test points (in blue box) were good. The condition of the remaining three points (in red box) was complicated, and we regarded them as constrained environments including big trees and tall walls that shelter the GNSS satellites signals significantly. The precise coordinates of these stations were computed by Bernese 5.0 software (the Astronomical Institute of the University of Bern, Bern, Switzerland). In these tests, GPS ONLY and GPS/BDS data were processed using the correction from the Hong Kong GNSS Network RTK Service Platform. To compose the weight matrix, the sigma of the carrier phase noise of BDS was set to 3 mm, the same as GPS. As a multipath of BDS code measurements is larger than GPS [9], the sigma of the code noise was set to 0.5 m for BDS, instead of 0.3 m as the GPS. The cutoff angle was set to 15°.

**Figure 7:**
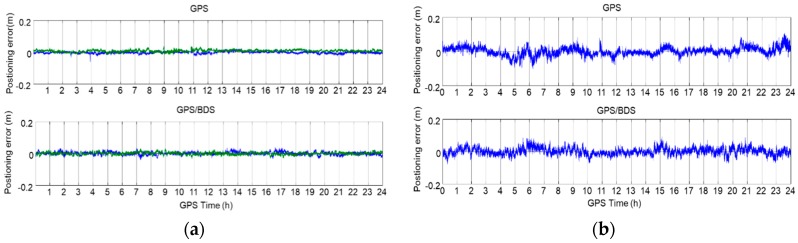
NRTK positioning error of the station at POLYU: (**a**) North (green) and East (blue); and (**b**) Up.

The positioning errors in the North, East, and Up directions of the station at sixth floor platform of Z core in POLYU are shown in Figure 7. Figure 7a shows the NRTK result of GPS ONLY, while Figure 7b shows the NRTK result of the GPS/BDS. From these figures, we can see that the GPS ONLY and GPS/BDS NRTK results were similar to each other. Table 1 shows the standard deviation (STD) of the positioning errors. The positioning accuracy was better than 1 cm in the North and East directions, and better than 2.3 cm in the Up direction. Furthermore, the positioning performance of GPS/BDS was slightly better than that of GPS ONLY. The NRTK STD of the positioning error in 24 h at the selected points, sub-grouped into two categories, good and constrained environments, are shown in Table 2, separately. In good environments, the NRTK positioning accuracy using the correction of the Hong Kong GNSS Network RTK Service Platform was 1–2 cm in the horizontal direction, and 2–4 cm in the vertical direction. In the constrained environments, the positioning accuracy was 2–3 cm in the horizontal, and 3–6 cm in the vertical directions.

### 4.3. Ambiguity Successfully Fixed Rate Test

To assess the ambiguity successfully fixed performance of the Hong Kong GNSS Network RTK Service Platform, four kinematic tests in a complicated environment were carried out in POLYU, Jordan, Sha Tin, and Hong Kong. The receiver of the rover station was Trimble R10 (Trimble Inc, Sunnyvale, CA, USA). GPS ONLY and GPS/BDS data were processed using the correction from the Hong Kong GNSS Network RTK Service Platform. The ambiguity-fix rate (AFR) was used to quantify the ambiguity successfully fixed performance with the following definition:

(15) $$\mathrm{AFR}=\frac{\mathrm{Number}\;\mathrm{of}\;\mathrm{epoch}\;\mathrm{with}\;\mathrm{fixed}\;\mathrm{solution}}{\mathrm{Total}\;\mathrm{number}\;\mathrm{of}\;\mathrm{epochs}\;\mathrm{observed}\;\mathrm{in}\;\mathrm{the}\;\mathrm{data}\;\mathrm{sets}}$$

where the Number of the epoch with fixed solution relied on the ambiguity validation method (R-ratio test).

For comparison, we calculated the AFR (Table 3) for the station at POLYU in Section 4.1. As shown in Figure 2, the observation condition of this station was very good. In Table 3, we can see that there were only 531 unfixed epochs for GPS. For GPS/BDS, all ambiguities were fixed. As a result, the AFR of GPS ONLY was 99.38%. For GPS/BDS, the AFR was 100%. For the convenience of the positioning result statistics, we built a GNSS data processing software for rover in this study. During the experiment, the rover stations received the GNSS data, and transferred the data to the server throng on the Internet. We used the software on the server to process the GNSS data to achieve the GNSS precise positioning. However, there would be data lost through the data transmission. As a result, the total number of epochs was not 86,400.

**Figure 8:**
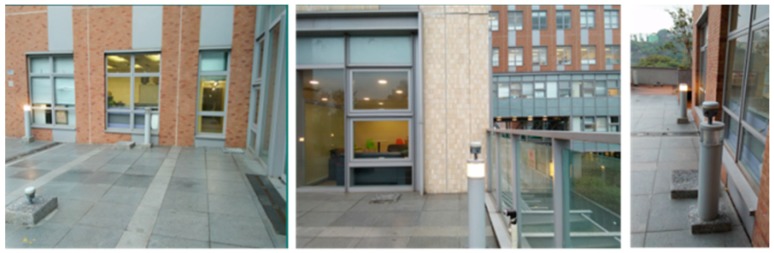
Experimental environment at POLYU.

**Figure 9:**
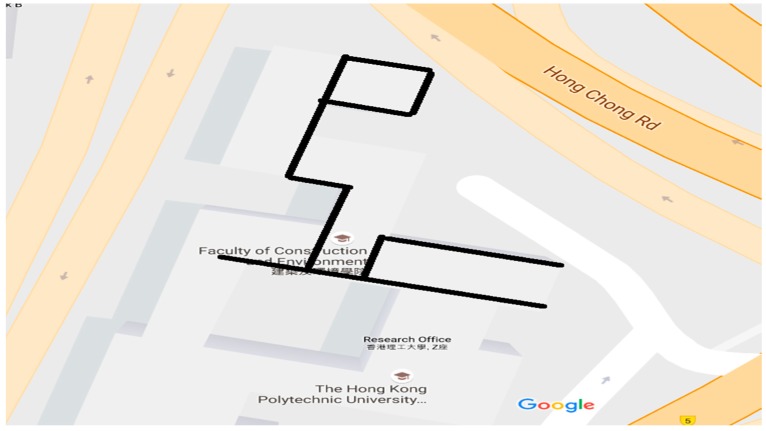
Experimental route at POLYU.

**Figure 10:**
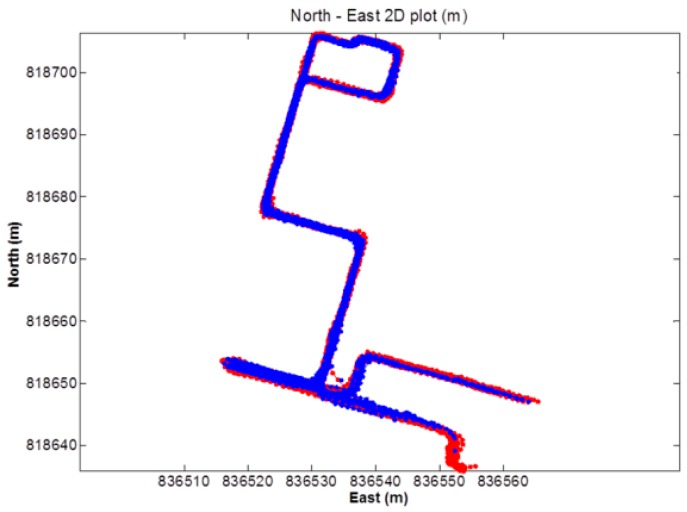
NRTK positioning result (red: GPS/BDS, blue: GPS).

The first kinematic test (walking test) in complicated environment was carried on the sixth floor platform of Z core in POLYU on 20 July 2016. Figure 8 and Figure 9 indicate the test environment and the experimental route, respectively. The test area was the terrace garden of the sixth floor where two walls will shelter the GNSS satellites signals. We walked repeatedly on a certain line for about 6 h with the RTK equipment. We sent and saved the original observations, and post-processed these observations to estimate the reference route. The GPS ONLY and GPS/BDS NRTK positioning results of the Hong Kong GNSS Network RTK Service Platform are shown in Figure 10. From these figures and the first column in Table 4, we can see that the AFR of GPS/BDS (red points in Figure 10) using the correction from the Hong Kong GNSS Network RTK Service Platform was 72.4%, which was much better than that of the GPS ONLY (32.4%, blue points in Figure 10).

**Figure 11:**
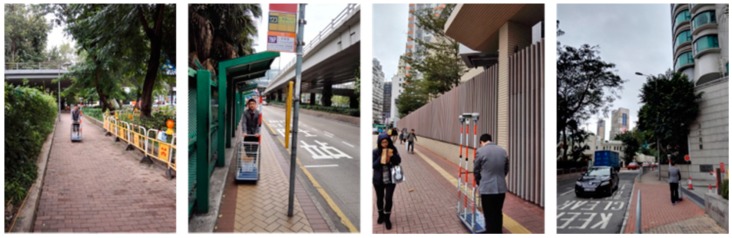
Experimental environment in HK Jordan.

**Figure 12:**
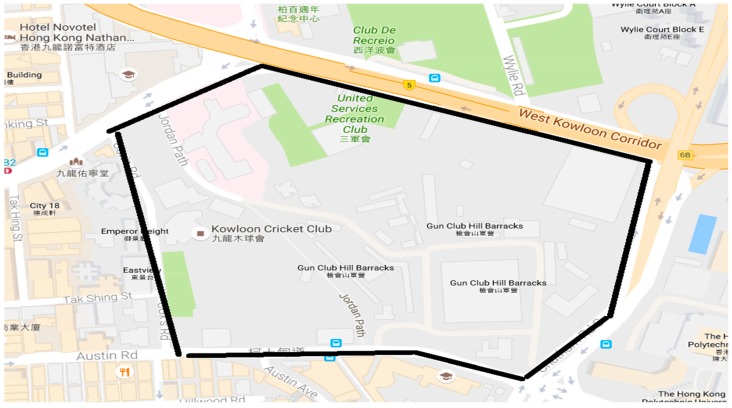
Experimental route in HK Jordan.

**Figure 13:**
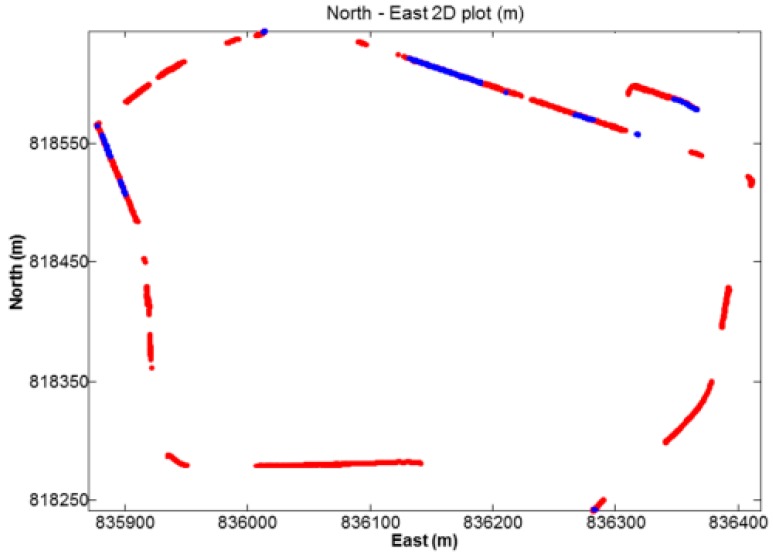
NRTK positioning result (red: GPS/BDS, blue: GPS).

The second walking test was carried in Jordan on 19 July 2016. Figure 11 and Figure 12 show the test environment and experimental route, respectively. As shown in Figure 11, the test environment was very complicated including big trees, bus stations, tall walls, and buildings, which would shelter the GNSS satellites signals significantly. The GPS ONLY and GPS/BDS NRTK positioning results of the Hong Kong GNSS Network RTK Service Platform are shown in Figure 13. From these figures and Table 4, we can find that the route of the GPS/BDS NRTK using the correction from the Hong Kong GNSS Network RTK Service Platform was clearer than that of the GPS ONLY NRTK. At the same time, the AFR of GPS/BDS NRTK was 53.2%. Again, it was much better than that of the GPS ONLY (19.1%).

**Figure 14:**
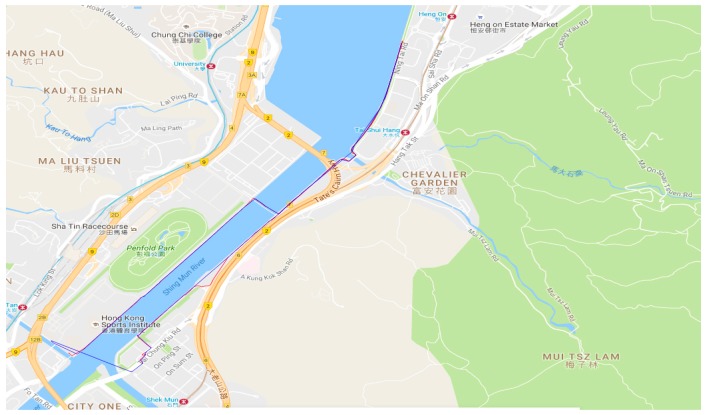
Experimental route in HK Sha Tin.

**Figure 15:**
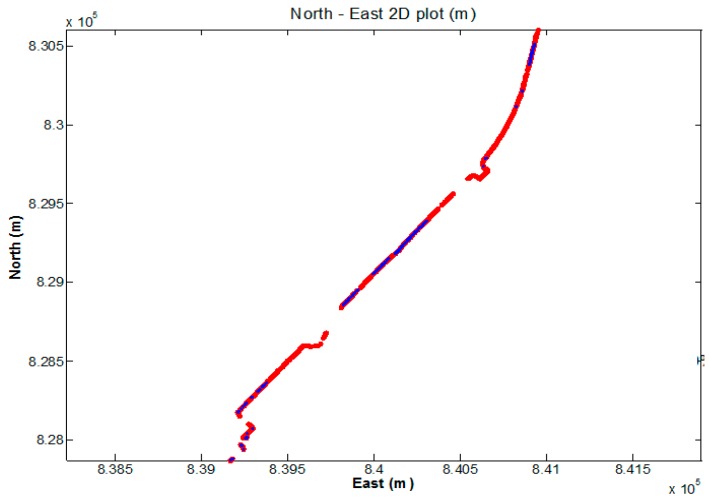
NRTK positioning result (red: GPS/BDS, blue: GPS).

The third walking test was carried out in Sha Tin on 26 July 2016. Figure 14 shows the experimental route, and there was a big river on one side of the route, and the other side had tall buildings, which would also significantly shelter the GNSS satellites signals. The NRTK positioning results are shown in Figure 15. From these figures and Table 4, we can see that the route of the GPS/BDS NRTK was clearer than that of the GPS ONLY. The AFR of GPS/BDS NRTK was 69.0%. Again, it was much better than that of the GPS ONLY (12.7%).

**Figure 16:**
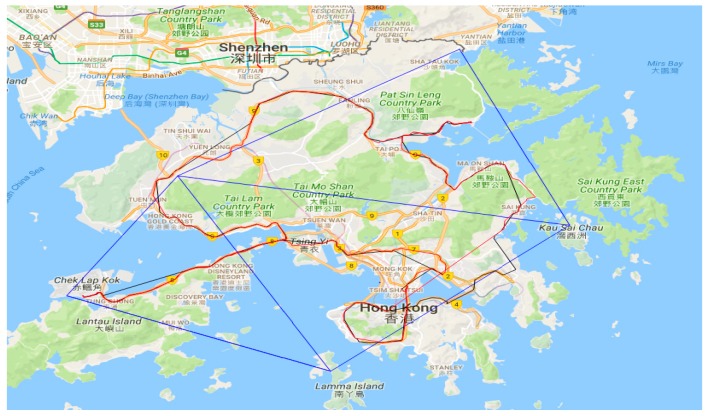
Experimental route in Hong Kong.

**Figure 17:**
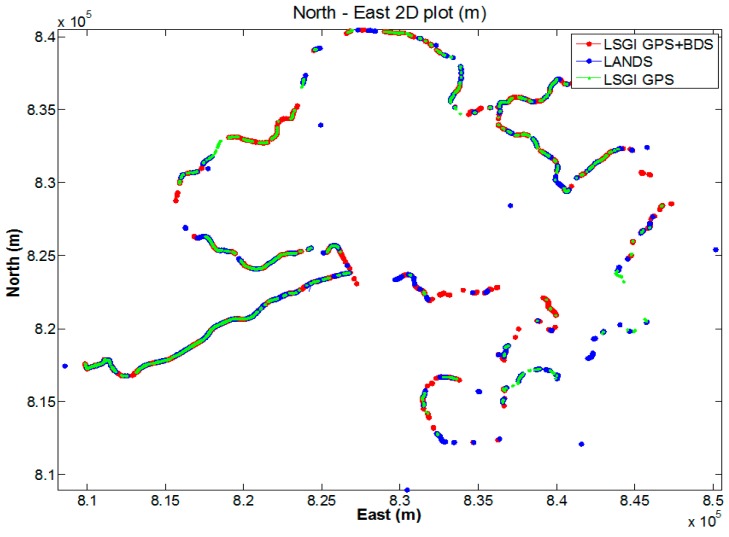
NRTK positioning result.

The last kinematic test was a car-driving test around Hong Kong. Figure 16 shows the experimental route. The NRTK positioning results are shown in Figure 17. The environment was very complicated and most of the GNSS signals were sheltered. However, the AFR of GPS/BDS using the correction from the Hong Kong GNSS Network RTK Service Platform was 33.4%, which was still much better than that of the GPS ONLY (24.2%). In this car-driving test, we also conducted the GPS NRTK test with the NRTK platform from the Lands Department, and the AFR of it was only 15.1%.

To sum up, the NRTK AFR in Hong Kong was better than 99% in good environment. In typical urban environment, the RTK positioning AFR with GPS/BDS with GPS/BDS was 33.4–72.4%, which was about 12.7–32.4% with GPS only.

### 4.4. Triple-Frequency GNSS RTK Test

Currently, there is still no triple-frequency BDS signal in the Hong Kong SatRef. To test the benefit of the triple-frequency signals of BDS on RTK, we set three GNSS triple-frequency receivers in Hong Kong. Two of them were located on the top floor of the Z core at POLYU. The other was near the HKFN station in Fanling. Twenty-four hours of GNSS observation of these three stations were collected on 6 October 2016, and the update rate was 1 s. Two baselines was chosen, one was the short-range baseline (between two stations at POLYU, 2.44 m), and the other was the medium-range baseline (between POLYU and HKFN station, 21.29 km). To examine the performance of the AR technique proposed in Section 3, both the proposed technique and the traditional method were employed to fix the ambiguities of the GPS triple-frequency signals. The scheme of the traditional method is: Firstly, get the float ambiguity solutions using the double difference triple-frequency pseudo-range and carrier phase observation (estimating the double difference ionospheric delay residual as parameters). Secondly, obtain the search space for the ambiguities adopting the LAMBDA method. Thirdly, fix the ambiguities with R-ratio test. This traditional method was also used in the AR for the dual-frequency signals. In data processing, we started from every epoch in the dataset until all ambiguities were fixed to their integers. Cycle slips were detected and repaired before AR processing was performed. The popular R-ratio (Equation (14)) was used as the ambiguity validation method and the threshold was set to 2.5.

**Figure 18:**
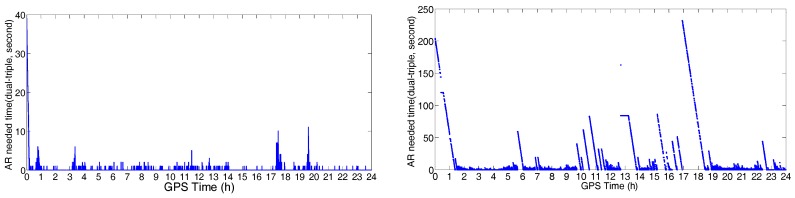
The AR required time difference between the dual-frequency RTK and triple-frequency RTK: short-range baseline, 2.44 m (**left**); and medium-range baseline, 21.29 km (**right**).

The difference of AR required time between the dual-frequency RTK and triple-frequency RTK (proposed AR technique) are shown in Figure 18. An average time of 1–2 s was needed to fix the ambiguity for the short-range baseline using both dual- and triple-frequency signals. For the medium-range baseline, triple-frequency RTK only needed 1.107 s to achieve the AR, and the dual-frequency RTK needed 22.914 s (mean time). Generally, the dual-frequency RTK required 0–250 s (initialization time) more than that of the triple-frequency RTK. The reason that the positioning performance of the triple-frequency model is better than that of the dual-frequency model would be: (1) the triple-frequency signals composing extra-wide lane and wide lane linear combinations, which will bring great benefit to the ambiguity resolution; (2) the triple-frequency signals increasing the number of the observations, which will improve the positioning accuracy; and (3) the triple-frequency signals strengthening the observation equation, and making the equation more robust. Table 5 shows the AR required mean time of the traditional method and the presented technique. For both the short-range baseline and the medium-range baseline, only 1.001–1.006 s were required for the AR of the EWL and WL (Table 5). For NL, the AR required time of the proposed technique in this study is slightly shorter than that of the traditional method in the short-range baseline. For the medium-range baseline, only 1.107 s is needed to fix the ambiguity using the proposed technique, which has significant improvement compared with that of the traditional method.

**Figure 19:**
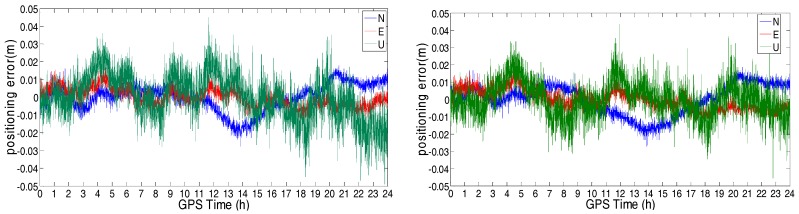
Positioning error of the dual-frequency (**left**) and triple-frequency (**right**) RTK for the short-range baseline (2.44 m).

**Figure 20:**
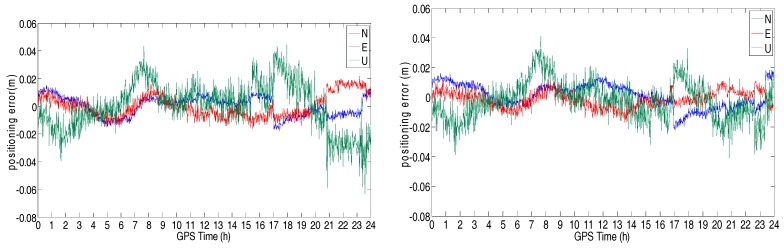
Positioning error of the dual-frequency (**left**) and triple-frequency (**right**) RTK for the medium-range baseline (21.29 km).

The positioning error in the direction of North, East, and Up are shown in Figure 19 and Figure 20. The STD of these errors is shown in Table 6. From these figures and table, we can see that the positioning errors of the triple-frequency singles were slightly smaller than that of the dual-frequency signals. The STD of the triple-frequency RTK was also better than that of the dual-frequency RTK.

## 5. Conclusions

In this study, the performance of GPS/BDS dual/triple-frequency NRTK in urban areas was tested under different modes (static, walking, and car driving mode) by using the Hong Kong GNSS Network RTK Service Platform we developed. The initialization time test, positioning accuracy test, ambiguity successfully fixed rate test, and triple-frequency BDS RTK test were carried out. Based on the discussions above, we obtained the conclusions as follows:

(1) The initialization time of the NRTK varied with the number of visible satellites and the quality of the observation.
(2) Centimeter-level NRTK service could be provided for users over Hong Kong by using the Hong Kong GNSS Network RTK Service Platform.
(3) In urban areas, GPS/BDS NRTK services for static, walking, and driving users significantly improved the ambiguity successfully fixed rate of NRTK service when compared with that using GPS signal alone. In typical urban environment, the RTK positioning ambiguity successfully fixed rate with GPS/BDS was 33.4–72.4%, which was about 12.7–32.4% with GPS only.
(4) The BDS triple-frequency observation significantly improved the initialization time and positioning accuracy of RTK in Hong Kong. For a baseline of about 20 km, the initialization time was reduced to 1 s with triple-frequency data, compared with 23 s with dual-frequency data.

## Figures and Tables

**Table 1:** STD of the positioning errors of the station at POLYU.

System	N(m)	E(m)	U(m)
GPS	0.009	0.008	0.023
GPS/BDS	0.009	0.007	0.021

**Table 2:** STD of the positioning errors of the seven test points.

Number of Points	Environment	Horizontal Direction	Vertical Direction
4	Good environment	1–2 cm	2–4 cm
3	Constrained environments	2–3 cm	3–6 cm

**Table 3:** AFR of the rover station at POLYU.

System	Number of Epoch	Number of Unfixed Epoch	POLYU
GPS	85575	531	99.38%
GPS/BDS	86264	0	100.00%

**Table 4:** AFR of the NRTK platforms.

	Walking Test			Car-Driving Test
System	POLYU	Jordan	Sha Tin	Hong Kong
GPS	32.4%	19.1%	12.7%	24.2%
GPS/BDS	72.4%	53.2%	69.0%	33.4%

**Table 5:** AR required mean time of the traditional method and the presented Technique (Unit: second).

2.44 m	EWL	WL	NL	21.29 km	EWL	WL	NL
Traditional Method			1.054	Traditional Method			19.09
Proposed Technique	1.001	1.003	1.003	Proposed Technique	1.003	1.006	1.107

**Table 6:** STD of the positioning errors and AR required mean time.

2.44 m	N(m)	E(m)	U(m)	21.29 km	N(m)	E(m)	U(m)
Triple-frequency	0.008	0.005	0.011	Triple-frequency	0.010	0.005	0.013
Dual-frequency	0.009	0.006	0.013	Dual-frequency	0.011	0.008	0.018
